# Antibiotikaresistenz – eine unendliche Geschichte?

**DOI:** 10.1007/s00103-026-04235-5

**Published:** 2026-04-30

**Authors:** Sebastian Günther, Peter Heisig

**Affiliations:** 1 Pharmazeutische Biologie, Universität Greifswald, Friedrich-Ludwig-Jahn-Str. 17, 17489 Greifswald, Deutschland; 2 Institut für Biochemie und Molekularbiologie, Pharmazeutische Biologie und Mikrobiologie, Universität Hamburg, Bundesstraße 45, 20146 Hamburg, Deutschland

In 2 Jahren jährt sich zum 100. Mal die Entdeckung des ersten Antibiotikums, Benzylpenicillin, durch Fleming. Die Aktualität sowie die gesundheitspolitische Relevanz und Dringlichkeit des Themas Antibiotikaresistenz sind weiterhin evident. Antimikrobielle Resistenzen stellen eine der größten Herausforderungen für die öffentliche Gesundheit dar.

Bereits Fleming war das Phänomen der Antibiotikaresistenz bekannt, wonach Bakterien bestimmter Arten abgetötet werden, während andere natürlicherweise überleben („primäre Resistenz“). Auch bei natürlich empfindlichen Bakterienarten fanden sich einzelne Stämme mit erworbener Unempfindlichkeit („sekundäre Resistenz“). Es entspann sich ein bis heute andauernder Wettlauf zwischen Bakterien und Mensch. Zahlreiche Strategien – darunter die Suche nach neuen antibiotisch wirksamen Naturstoffen, deren partielle chemische Modifikation, die Synthese rein chemischer Wirkstoffe sowie Kombinationstherapien mit Antibiotika und Resistenzinhibitoren – zeigten jedoch meist nur vorübergehende Effekte. Aufgrund genetischer Variabilität durch Mutationen und Gentransfer konnten sich rasch neue Resistenzmechanismen ausbilden und verbreiten.

Den Auftakt dieses Themenheftes bildet eine Übersicht von *Heisig und Heisig* zu den Grundmechanismen der Entstehung und Verbreitung von Resistenzgenen. Viele Resistenzgene entstammen ursprünglich Antibiotika-produzierenden Bakterien, in denen sie als Schutzmechanismus dienen, und sind auf pathogene Bakterien übertragbar. Begünstigt durch hohe Mutationsraten und schnelle Vermehrung zu hohen Zellzahlen können unter einer inadäquaten Therapie aber auch neu entstandene Resistenzeigenschaften selektiert werden. Unter Selektionsdruck – etwa durch suboptimalen Einsatz von Antibiotika – können sich solche Mutanten rasch in Populationen vermehren.

Obwohl Antibiotikaresistenzen seit vielen Jahren Gegenstand intensiver wissenschaftlicher und gesundheitspolitischer Diskussionen sind und im *Bundesgesundheitsblatt* wiederholt thematisiert wurden (z. B. in den Ausgaben 5/2018, 6/2023 und 6/2025), rechtfertigen neue Entwicklungen ein weiteres Themenheft. Während frühere Beiträge vor allem die epidemiologische Situation, Surveillance-Systeme, Antibiotikaverbrauch sowie One-Health-Übertragungswege beleuchteten, erweitert das vorliegende Heft die Perspektive um Faktoren, wie den anthropogenen Eintrag von Antibiotikarückständen in Umweltkompartimente, neue diagnostische und datenbasierte Ansätze, Verhaltensfaktoren beim Antibiotikaeinsatz sowie innovative Strategien zur Resistenzvermeidung und -umgehung. Damit spiegelt das Themenheft eine inhaltliche Verschiebung wider: von der primären Beschreibung des Resistenzproblems hin zu einem stärker integrativen Verständnis von Ursachen, Einflussfaktoren und konkreten Interventionsmöglichkeiten entlang der gesamten One-Health-Kette.

Bei der Resistenzentwicklung ist ein solcher Selektionsdruck hervorzuheben, der durch übermäßigen und unsachgemäßen Einsatz von Antibiotika erzeugt wird. Dieser ist nicht nur ein klinisches Phänomen: Antibiotikarückstände werden zunehmend auch in Umweltkompartimenten wie Böden, Gewässern und Abwässern nachgewiesen, teilweise in Konzentrationen, die als besorgniserregend gelten und ebenfalls zur Selektion resistenter Mikroorganismen beitragen können. Der Beitrag von *Prüß und Guenther *zu Antibiotikaresistenzen in der Umwelt durch Eintrag von Antibiotikarückständen beleuchtet diesen ökologischen Aspekt. Außerdem werden Antibiotika auch bei Tieren, nicht nur klinisch in der Tiermedizin, sondern auch in niedrigen Konzentrationen in der Tierhaltung eingesetzt. Über Ausscheidungen können dabei Konzentrationen entstehen, die ebenfalls einen Selektionsdruck auf pathogene Erreger und Umweltkeime ausüben. Der Artikel von *Flor und Tenhagen* zur Entwicklung von Antibiotikaanwendung und -resistenz in der Tierhaltung beleuchtet die Maßnahmen zur Reduktion der Antibiotikaresistenz und deren Ergebnisse am wichtigen Beispiel von Mastgeflügel.

Angesichts der zunehmend zeit- und kostenintensiven Anforderungen an die Zulassung neuer Antibiotika sind die Aufwendungen für die oben angegebenen Strategien gegen Resistenzentwicklung stark angestiegen. Vor dem Hintergrund der stagnierenden Entwicklung neuer Antibiotika zielen diese Maßnahmen darauf ab, die Resistenzentwicklung zu reduzieren und die Wirksamkeit vorhandener Antibiotika möglichst lange zu erhalten. Hierzu wird eine Reihe organisatorischer und technischer Maßnahmen eingeführt bzw. optimiert und neue Wege in der Verfügbarmachung alternativer, z. B. biologischer Therapieoptionen beschritten.

Ein besonders wichtiger Aspekt bei der Entwicklung von Antibiotikaresistenzen ist dabei die Selektion im klinischen Umfeld, z. B. im Körper eines Patienten oder bei Ausbrüchen eines resistenten Erregers innerhalb einer Station. Die Entscheidung für eine adäquate, rationale Antibiotikatherapie erfordert wichtige Basisdaten über Identität und Antibiotikaempfindlichkeit des verursachenden Erregers. Hierfür ist eine sichere und möglichst zeitnahe Information aus dem medizinisch-mikrobiologischen Labor essenziell. Die derzeit noch vorgegebene Standardmethode weist Wachstumshemmung nach und benötigt mindestens 12 h. Moderne genetische Techniken (z. B. PCR (Polymerase-Ketten-Reaktion zum Vervielfältigen von DNA), DNA-Sequenzierung) lassen diese Zeit auf wenige Stunden reduzieren, sind aber nicht in jedem Fall einsetzbar. Neben technischen Weiterentwicklungen sind vor allem die Vereinheitlichung der Verfahren sowie die Aussagekraft der Daten wichtig. Diese Thematik wird im Beitrag von *Berinson et al.* über die integrierte Diagnostik antimikrobieller Resistenzen diskutiert.

Ebenso hilfreich, z. B. für die landes- oder europaweite Entwicklung und Verbreitung von Antibiotikaresistenzen sind Daten aus epidemiologischen Studien, die nach unterschiedlichen Gesichtspunkten (Erreger, Therapie, Infektionskrankheit) Aussagen über den effektiven Einsatz von Antibiotika, insbesondere auch als kalkulierte Initialtherapie, zulassen. Im Beitrag von *Eckmanns et al.* zum Thema Entwicklung der Epidemiologie antibiotikaresistenter Erreger in der Humanmedizin werden solche Daten vorgestellt und im Hinblick auf bisherige und noch angestrebte Erfolge die Bedeutung nationaler und internationaler Strategien wie Surveillance-Programme, rationaler Antibiotikaeinsatz (Antibiotic Stewardship – ABS), verbesserte Hygiene sowie One-Health-Ansätze bewertet mit dem Ziel, eine deutliche Reduktion von infektionsbedingten Todesfällen zu erreichen. Gleichzeitig wird der Bedarf an neuen therapeutischen Strategien, verbesserter Diagnostik sowie verstärkter Forschung und politischen Maßnahmen hervorgehoben.

Antibiotika nehmen unter pharmazeutischen Wirkstoffen eine Sonderstellung ein, da ihr Angriffsziel nicht Teil menschlicher Zellen, sondern bakterieller Erreger im menschlichen Körper ist. Im Vergleich zu konventionellen Arzneimitteln weisen Antibiotika zudem spezifische pharmakokinetische und pharmakodynamische Eigenschaften auf, da ihre Wirksamkeit nicht nur von der Konzentration im Körper, sondern auch von der Interaktion mit der Zielpopulation der Bakterien abhängt. Nebenwirkungen betreffen daher nicht nur den Menschen selbst, etwa durch unerwünschte Arzneimittelwirkungen, sondern auch bakterielle Gemeinschaften wie die menschliche Mikrobiota, deren Zusammensetzung und Funktion durch Antibiotikatherapien verändert werden kann. Die Resistenzentwicklung ist Teil dieses Gesamtbildes, da Antibiotika Selektionsdruck auf bakterielle Populationen ausüben. Der Beitrag von* de With et al.* über rationalen Antibiotikaeinsatz auf der Basis von ABS widmet sich dieser Thematik und stellt Voraussetzungen für eine zielgerichtete Therapie vor, unter Bewertung der jeweiligen Konsequenzen.

Weiterhin zeigen Antibiotika bei ihrer Passage durch verschiedene Teile des menschlichen Organismus auf dem Weg zu ihrer Zielstruktur in einem Bakterium komplexere pharmakokinetische Eigenschaften. Insbesondere Patienten mit altersbedingt von „durchschnittlichen Erwachsenen“ abweichenden Eigenschaften wie Kinder oder ältere Menschen benötigen häufig eine besondere Anpassung der Antibiotikaanwendung, z. B. bezüglich der Dosis. Der Beitrag von *Hübner* stellt die dabei bestehenden Probleme dar und betont die Wichtigkeit fundierter Kenntnisse der Behandelten zu pharmakologischen Besonderheiten dieser Gruppen sowie einer guten Aufklärung und Begleitung der Patienten.

Aber auch die Rolle des Patienten als eigentlicher Mittelpunkt der Antibiotikaanwendung wird beleuchtet. Betrachtet werden dabei psychologische Faktoren in der Einstellung eines Patienten zur Antibiotikatherapie, die im Vergleich zu anderen Arzneimitteln nicht nur positive, sondern auch ängstliche negative Einstellungen hervorrufen können. Im Beitrag von *Böhm et al.* werden psychologische Einflussfaktoren auf das Einnahmeverhalten des Patienten und damit auf eine Begünstigung von Resistenzentwicklung sowie mögliche Verhaltensweisen des behandelnden Arztes in der Beziehung zum Patienten dazu vorgestellt.

Neben klassischen Antibiotika rücken auch alternative therapeutische Strategien, insbesondere biologisch basierte Therapien, zunehmend in den Fokus. So ist der Einsatz von Bakteriophagen – Viren, die ausschließlich Bakterienzellen infizieren und diese direkt oder nach einem Zwischenaufenthalt in den Zellen abtöten – schon seit über 100 Jahren bekannt und wird in Teilen Osteuropas und Russlands seitdem zur Infektionsbehandlung verwendet. Aufgrund ihrer Fähigkeit zur Vermehrung in Bakterienzellen können Phagen sich bei Entwicklung einer Phagenresistenz ihrerseits genetisch so anpassen, dass sie wieder die Fähigkeit zum Abtöten der Bakterien erlangen. Der Beitrag von *Heisig und Heisig* untersucht, wie sich eine kombinierte Therapie mit Antibiotika und Phagen auf die Empfindlichkeit und Resistenz bakterieller Infektionserreger auswirkt. Ein anderer biologischer Ansatz beruht auf der Modifikation des natürlichen Mikrobioms zur Eradikation multiresistenter Erreger, der im Artikel von *Weirauch et al.* beschrieben wird. Am Beispiel des komplexen Darmmikrobioms, für das ein hohes Risiko der Besiedlung durch multiresistente Erreger besteht, werden Möglichkeiten zur Reduktion/Elimination resistenter Erreger vorgestellt und die klinischen Optionen bewertet. Einen neuartigen Ansatz stellt schließlich der Einsatz von modernen Impfstoffen (rekombinant, reverse oder mRNA) im Beitrag von *Klimka und Maus *über Möglichkeiten moderner Impfstoffe zur Prävention bakterieller Antibiotikaresistenz dar. Bisherige Erfahrungen, aber auch Aspekte wie die Akzeptanz bei Patienten werden vorgestellt und bewertet.

In ihrer Gesamtheit verdeutlichen die Beiträge dieses Themenhefts, dass Antibiotikaresistenzen nicht allein ein mikrobiologisches oder klinisches Problem darstellen, sondern durch ein komplexes Zusammenspiel biologischer, epidemiologischer, ökologischer und gesellschaftlicher Faktoren geprägt sind. Ein wirksamer Umgang mit antimikrobiellen Resistenzen erfordert daher ein integriertes Verständnis dieser Zusammenhänge sowie koordinierte Maßnahmen. Eine Übersicht auf unterschiedlichen Ebenen über solche Maßnahmen, ihre Bedeutung, Erfolge und – noch – Defizite soll die vorliegende Ausgabe des *Bundesgesundheitsblattes* liefern. Verbunden damit ist es aber auch, die Bereitschaft und das Engagement zu wecken, durch breiten klinischen und wissenschaftlichen Erfahrungsaustausch die globale Problematik „Antibiotikaresistenz“ gemeinsam anzugehen und für Patienten verständlich und dadurch akzeptabel umzusetzen.

Im Namen aller Autorinnen und Autoren wünschen wir interessante Eindrücke und viel Freude bei der Lektüre.

